# Saudi Nurses’ Competency Learnings and Experiences from the Newly Developed Advanced Nursing Practice Diploma Program in Saudi Arabia: A Phenomenological Study

**DOI:** 10.7759/cureus.7584

**Published:** 2020-04-07

**Authors:** Khalid Abdullah S Aljohani

**Affiliations:** 1 Community Health, Nursing College-Taibah University, Medina, SAU

**Keywords:** advanced nursing diploma, competency, clinical learning environment, nursing education, saudi arabia, qualitative

## Abstract

Introduction

In response to Saudi Vision 2030, the Saudi Commission for Health Specialties has taken the lead in supporting healthcare system development by providing advanced nursing practice training diplomas. The aim of this study is to explore the experience of nursing trainees during their enrollment in the newly established Saudi Commission for Health Specialties (SCFHS) Advanced Nursing Practice (ANP) diplomas.

Methods

We employed a descriptive phenomenological approach in this study. Collaizi’s distinctive process was utilized as a guide for the data analysis of 12 interviews.

Results

Exploring the participants’ experiences revealed four themes: (1) the organizational culture valuing the learner and their learnings; (2) the commitment to continuous quality care improvement; (3) challenges in the trainer-trainee relationship; and (4) the vague career track of the trainees.

Conclusion

The identified challenges faced by the nursing trainees may hinder the optimum utilization of these program outcomes. Interventions to overcome the identified challenges should be initiated by all stakeholders.

## Introduction

The development of the nursing profession in the Kingdom of Saudi Arabia (KSA) is one of the foci of the country’s Vision 2030. The vision includes an emphasis on the need to increase the number of well-equipped Saudi nurses as well as on raising standards of education and training for them [1]. Strategies employed by the country include horizontal and vertical expansion of nursing education programs through strengthening the bachelor’s program and postgraduate degree while also offering Advanced Nursing Practice (ANP), with the latter considered to be a nursing specialty diploma.

ANP is an aspect of the continuing professional development that emerged in the KSA and encompasses growth and development in holistic knowledge, skills, and the attitude of Saudi nurses in focusing on their nursing profession and nursing care. The Saudi Commission for Health Specialties (SCFHS) constantly drives reforms within the healthcare system of the country through the mobilization of efforts to advance the realm of professional nursing education, to recognize the existence of and address the challenges faced in the nursing profession, and to promote specialty programs to further train Saudi nurses to become more skillful and efficient professionals.

In 2014, one of the challenges faced by Saudi nurses was an increased demand for nursing services due to limited advanced nursing education opportunities [2]. Nurses' training was limited to the training that they had completed during their baccalaureate degrees. Unlike in other countries, specialization in the nursing profession was not thought of as a priority; hence, nurses just stagnated as generalists. This limitation resulted in poor satisfaction in nurses’ personal and professional dimensions [2,3]. This research study explores the lived experiences of ANP diploma trainees regarding the learning opportunities and challenges they face.

## Materials and methods

Research design

This study utilized a qualitative study design following a descriptive phenomenological approach to achieve a deeper understanding of the phenomenon. In addition, it enabled the researcher to capture the richness of the explored area of information based on the participants’ descriptions of real-life experiences [4].

Study participants

This research study was conducted in two selected Ministry of Health (MOH) tertiary training hospitals in the KSA. The study involved 12 newly qualified Saudi nurses as participants. The inclusion criteria for this study included the following: Nurses who had enrolled in the ANP diploma, had passed the first year of the diploma program, were currently trainees in government healthcare centers, and were willing to participate in the study. Data saturation was achieved with the 10th participant; however, the researcher still interviewed two additional participants to further enrich the data and to assess whether new information would still surface.

Data collection procedure

Upon securing the approval of the ethics committee and hospital administrators as well as the consent of the participants, data gathering commenced. Face-to-face, in-depth, semi-structured interviews were conducted, which were audiotaped and transcribed by the researcher. In addition, interviews and field notes were recorded, which were utilized during data analysis. Each interview session lasted 40-60 minutes, with a mean duration of 47 minutes. The study was conducted between October and December 2018. The interview protocol included information for the participants, their consent to participate in the study, and the researcher’s contact details in case the participants had an inquiry after the interview. In addition, the interview guide included open-ended questions to explore trainees’ experiences relating to their journey in completing the ANP diploma and any potential challenges and opportunities related to their learning development.

Data analysis

Collaizi’s distinctive process was used to provide rigorous data analysis [5,6]. The process included: (1) familiarizing with the interview transcript to grasp the general ideas of the participants; (2) identifying significant words and phrases from the participants’ experiences through highlighting them as essential cues; (3) formulating meanings from the significant statements by repeatedly listening to the audio recording in part and as a whole while reading the transcript; (4) clustering the cues with the same meaning and coding them; (5) developing themes by elaborating on the exhaustive descriptions of the experiences of the ANP diploma trainees; (6) producing the fundamental structure of participants’ experiences through condensing exhaustive descriptions and substantiating them with literature; and (7) seeking verification of the results of the analysis through presenting them to the participants.

## Results

Demographic characteristics

The study sample consists of four female and eight male nurses who are Saudi nationals. All of them were previously employed in the governmental healthcare sector except for one nurse who was a fresh graduate and unemployed. The mean number of years of employment for the participants was 8.75 years.

Data findings

Utilizing Colaizzi’s method of analyzing data on the learning opportunities and challenges within the realm of ANP experiences of the participants of this research study, four themes emerged: (1) the organizational culture valuing the learner and their learnings; (2) the commitment to continuous quality care improvement; (3) challenges in the trainer-trainee relationship; and (4) the vague career track of the trainees.

Theme I. The Institutional Culture Valuing the Learner and their Learnings

The ANP diploma trainees were asked about their experiences during their initial months of training in the institution as well as in their didactic sessions. All the trainees shared that appropriate series of orientations had been conducted by the healthcare institutions for them to become more familiarized with the diploma program and the specialization they were training in. Some of the comments shared were as follows (P refers to participants):

P8: “During the orientation in the first two weeks of the diploma, we learned about infection control practices, read the diploma booklet, and visited the training center departments.”

P10: “We were given sufficient time to become familiar with the hospital policies and regulations …and with what training diploma we were specializing in. It is important to receive orientation so that we are guided on what to expect while we are here.”

The participants also shared how the healthcare institutions invited external expert lecturers from various universities in the country to further enrich the training experience.

P6: “…epidemiology, biostatistics, and research were delivered by the university staff, which gave us the confidence to understand the components of the module.”

P3: “We love learning from the experiences and lectures of guest professors from universities. It is a good experience for all trainees.”

Theme II. The Commitment to Continuous Quality Care Improvement

It was observed from the participants’ reactions and perceptions that they had enjoyed dynamic training and lecture approaches, such as the use of flipped, hands-on case studies and inter-professional learning environments while studying. All the participants agreed that the horizon of their learning widens when they are taught in not just the traditional way and when other allied health professionals enter the scenario of teaching and learning.

P2: “The diploma supervisor asked us to prepare and present the topics of many modules and have group discussions to cover missing information or stress important clinical issues.”

P3: “We have been taught by a consultant physician. It was a totally different experience. We benefited from his lectures, which were based on experience and clinical knowledge.”

For the continuous improvement of the trainees, the training centers regularly conducted spot checks and formal and informal evaluations.

P1: “The supervisor and clinical instructor monitor our nursing care by holding individual and group conferences, bedside conferences, and peer interviews. They also give us feedback.”

Theme III. Challenges in the Trainer-trainee Relationship

Coupled with the vast learning opportunities of the participants are the challenges that they faced and still encounter while enrolled in the ANP diploma program. Among the most common challenges that they encountered were the inability of the clinical instructor to address their inquiries, insufficiency of clinical instructors, specialization interest versus training mismatch, the non-involvement of trainees in committee meetings concerning them, and a lack of learning resources and facilities. Nine of the participants shared the following experiences:

P1: “The staff stress that they have no time to respond to the trainees’ inquiries …and they just ignore us.”

P4: “No Saudi nationals were involved in the training process, which I think made it harder for us to communicate with them.”

Related to their enrolled specializations, six of the participants expressed the following views:

P7: “Some of the trainees were interested in another specialization; however, we were forced to take the specializations they offered us. We had no choice.”

P4: “The workplaces reject any specialization that does not match the organizational scope of practice …these requirements give no option except to study the specialization they offer.”

Regarding communication and involvement issues of the participants in meetings, eight of them commonly shared that they have no voice to speak about what they need and feel. With regard to the lack of learning resources, all participants shared their concerns about the lack of a discussion room and learning simulators:

P4: “Although a classroom was allocated to us [trainees] earlier in the diploma, the training center education department uses the classroom for physicians’ educational activities, which in some cases gives us no other option except to use unappropriated spaces, such as patient rooms, for our lectures or case presentations.”

P9: “…there is one simulation room, but we do not use it.”

Theme IV. The Vague Career Track of the Trainees

In as much as Saudi Vision 2030 stipulates a raised standard in nursing education, training, and care, the participants were still unsure of what their professional role and future would be after completing the diploma due to some unanswered questions that they had:

P7: “We are not sure about what our title and role will be after graduating with the diploma …also, why not offer such a diploma at a university, such as a master of nursing?”

P12: “The diploma certificate is not respected enough to encourage Saudi nurses to enroll. Studying a master’s degree in nursing is a better option, considering my experience with this diploma.”

## Discussion

Our analysis of the lived experiences of the ANP diploma trainees regarding their learning opportunities and challenges while enrolled in a specific diploma focused on specialization yielded four significant themes. The vague career track of the trainees' theme brought to surface the obvious realm of their learning experiences as nurses engaged in continuous professional development. Further, issues and concerns were shared by the participants, which are eye-openers for diploma implementers, institutional training facilities, and the trainees to resolve in order to produce more globally competent Saudi nurses.

This research study presents how the success of the ANP diploma needs constant orientation and guidance so that the trainees are continuously guided and motivated. Orientation is a crucial stage in role transitions, such as in specializations, role socialization, and the sense of well-being among nurses, and a positive correlation should exist between conducting holistic orientations and nursing practice role transitions [7,8,9]. Also, the conduct of such initiatives affects the psychological preparation of nurses for acquiring new roles in advancing nursing care and optimizing outcomes [10,11]. Further, the involvement of experts in sharing their knowledge and skills plays a crucial role in building stronger foundations and fostering greater cooperation between the training institution and the trainees. This result of the study supports the concept that the unique opportunity for ANP diploma trainees to acquire an inter-professional mindset to gain skills in socialization and learning is a means of providing more patient-centered care through future certified nurse specialists [12]. In addition, advanced or specialized nursing knowledge and skills should be delivered by experts with a strong background in teaching to ensure that such knowledge and skills are transferred in a safe, evidence-based environment [13]. As such, this research study supports a multidisciplinary approach in order to equip Saudi nurses with the necessary competencies.

Another major result of this research study relates to the commitment to continuous quality care improvement through dynamic training and didactic strategies and an inter-professional learning approach. In order to train future certified nurse specialists and to decrease the rendering of fragmented care services, interprofessional education has been constantly emerging to enrich the social dimension, communication skills, respect, and trust of ANP diploma trainees. Interprofessional learning facilitates transitions in the roles of nurse generalists through enhancing their competency and establishing a supportive workforce and thereby reducing stress [14].

In spite of the learning opportunities that the ANP diploma training opens up, trainees also face a lot of challenges, which affect their professional growth and development. The trainees gain a sense of independence, considering that they are already engaged in their respective specializations, and enhanced supervision contributes to a more effective outcome, especially because the ANP diploma is new in the country [7]. Being unsure as to whom to approach for clarifications and/or being unable to receive a proper response hinder the trainees’ achievement of diploma goals [15].

Other challenges commonly shared by most of the participants were the interest and training mismatches, as well as the lack of seats for trainees during committee meetings concerning them and the lack of facilities for holding discussions. They were forced to enroll in a specialization that was not their area of interest, and arising policies were merely cascaded to them, leaving them no choice but to adhere to those. This should be addressed by the implementers and accredited training institutions if they want to produce highly competent Saudi nurse specialists. Looking through the lens of Vision 2030 at improving and raising the standards of training among Saudi nurses, the trainees must be given opportunities to participate in health plans and quality initiatives to regulate and govern their ANP diploma experience [1]. In order to drive transformation among local nurses, the trainees enrolled in the diploma must have a secure healthy environment where open communication occurs. The latter aids in the realization of empowering, strengthening, and further honing of their skills and knowledge in nursing.

From an intrapersonal stand, the trainees’ question what their roles will be after completing the diploma specialization they chose. This theme is an alarming facet as this significantly implies that the trainees are not fully aware of the scope of the responsibilities, or practice as a whole, of their chosen specializations. In the insufficiency or even the absence of full awareness about the clearly defined scope of nursing specialization practice, it would be really challenging to delineate their roles and responsibilities [16]. It is in this aspect of the training that the KSA’s initial effort to realize its Vision 2030 for healthcare is still insufficient for upgrading nursing education and continuous professional development diplomas through the ANP diploma, both horizontally and vertically.

This research study is grounded in Vision 2030 and on the learning opportunities and challenges relating to the ANP diploma trainees, which reflect how Saudi nurses are embracing the privilege and opportunity to transform and uplift their chosen profession. They are prepared to face the challenge of professional learning and upgrade their nursing care competencies. At the same time, the results of this study revealed that the ANP diploma must be more regulated in such a way that policies relating to the diploma should have its wrinkles ironed out. In this way, trainees will have a clearer scope of practice and roles. From another perspective, Vision 2030 is still in its infancy since it was only fully introduced in the year 2017. Improvements to health-related stipulations can still be made, based on scientific evidence.

Based on the findings of this study, our recommendations deemed necessary for the improvement of the ANP diploma in the country towards the attainment of Vision 2030 for healthcare are: partnerships with national universities should be formed to strengthen efficient execution of the program; the country’s immediate need for a specialized nursing workforce should be identified, and measures should be taken to ensure that programs stick to strict schedules and timeframes; avoid mismatches and forced enrollment in specializations in which the trainees are not interested; all training center staff who participate in training should undergo preceptorship training; empower trainees' voices; and provide structured career counseling.

## Conclusions

This research study concludes that ANP diploma trainees are able to increase their competency when they are properly oriented and guided by experts in education and allied health professionals, and when they acquire the required clinical skills through various training strategies. On the other hand, the trainees encounter issues when there is an insufficiency of supervising clinical instructors, a mismatch in interest versus training offerings, a lack of a voice on their part for improving the training experience, and a lack of learning and training facilities.
